# Lower Extremity Osseointegration Postoperative Rehabilitation Protocols: A Scoping Review

**DOI:** 10.1093/ptj/pzae139

**Published:** 2024-10-10

**Authors:** Matan Grunfeld, Taylor J Reif, S Robert Rozbruch, Jason S Hoellwarth

**Affiliations:** School of Medicine, New York Medical College, Valhalla, NY, United States; Osseointegration Limb Replacement Center, Hospital for Special Surgery, New York, NY, United States; Osseointegration Limb Replacement Center, Hospital for Special Surgery, New York, NY, United States; Osseointegration Limb Replacement Center, Hospital for Special Surgery, New York, NY, United States

**Keywords:** Amputation, Lower-Extremity, Osseointegration, Rehabilitation

## Abstract

**Objective:**

Lower-extremity transcutaneous osseointegration is a rehabilitation alternative to socket-suspended prostheses. The rehabilitation process, philosophies, and routines remain under-described. This review, primarily, identifies commonalities and differences among protocols. Secondarily, strategies are proposed to streamline future research of post-osseointegration surgery rehabilitation.

**Methods:**

Two differently-phrased queries of Google Scholar, Pubmed, Embase, and Web of Science were performed. First using either “osseointegration” or “osseointegrated” or “bone anchored prosthesis” AND [last name]. Second, replacing author name with “physical therapy” or “rehabilitation”. Six hundred eighty-eight articles were identified describing lower-extremity rehabilitation following osseointegration. Following software-based deduplication, manual abstract and full-text review, article reference evaluation, and use of Google Scholar’s “Cited by” feature, 35 studies were fully analyzed. First, a consolidated summary was made of protocols focusing on stages, timing, and other descriptions of postoperative rehabilitation. Subsequently, strengths and limitations of protocols were considered to propose potential strategies to investigate and optimize postoperative rehabilitation.

**Results:**

All articles describe rehabilitation having this same order of goal progression: from surgery to gradual weight bearing and final goal of independent ambulation. The most impactful difference influencing the stated final goal of independent ambulation was whether one or two surgical stages were performed. No articles reported patient success rate achieving proposed goals and timing, or challenges during rehabilitation. Therefore, the first research suggestion is to investigate actual success rates achieving proposed goals and timing. Second, to further explore rehabilitation of performance deficits, beyond unaided ambulation. Finally, to incorporate technology such as mobility trackers to more objectively understand prosthesis use and mobility.

**Conclusion:**

All lower-extremity osseointegration rehabilitation literature recommends identical goal progression order. No studies evaluate patient challenges or variation. Understanding and addressing such challenges may enhance postoperative rehabilitation.

**Impact:**

This article consolidates published rehabilitation protocols post-osseointegration surgery. Specific analysis and experimentation of the protocols may enhance the uniformity and potential of patient rehabilitation.

## INTRODUCTION

Lower extremity amputation (LEA) presents a formidable rehabilitation challenge for patients. The reduced mobility and need for a prosthesis often hinder the patient’s quality of life (QOL) compared to a normally functioning leg. The prevalence of LEA is substantial: in the United States, an estimated 150,000 people undergo LEA annually.^1–3^ Currently, prosthetic limbs most commonly suspend from the residual limb via a mechanical or atmospheric mechanism, which sucks or squeezes the skin into a socket. This skin-socket interface is associated with challenges that contribute to the poor mobility and QOL: skin irritation, excessive sweating, difficulty walking at high pace or on unpaved surfaces, and pain, which results in periodic and/or prolonged periods of prosthesis disuse for many patients.^4–7^

An alternative rehabilitation option is transcutaneous osseointegration following amputation (TOFA).^2^^,^^8^ This is a surgical reconstruction which provides a permanent, skeletally anchored, transcutaneous metal implant, onto which many terminal prosthetic devices can be attached. Outcome reviews identify that a large majority of patients experience mobility and QOL benefits versus a socket prosthesis.^9^ However, the rehabilitation process, philosophies, and routines following TOFA are minimally-described. Substantial differences among protocols may cause confusion or apprehension among clinicians and patients. Further, the lack of specific rehabilitation strategies for patients who have undergone TOFA may limit patient benefit, given that patients with LEA using socket prostheses benefit from specific guided therapy to maximize recovery.^10^^,^^11^

To address this knowledge deficit, this scoping review of lower extremity rehabilitation following TOFA surgery was performed, with the specific intent to present an organized consolidation of existing recommendations. The primary aim was to identify commonalities and differences among protocols. The secondary aim was to suggest themes and focus areas which may best streamline future TOFA rehabilitation research. It is emphasized that this scoping review does not aim to rank or compare protocols regarding their appropriateness, success, or popularity; only to present the spectrum of principles.

## METHODS

Institutional review was not necessary to conduct this study.

### Search Strategy

The literature review software Rayyan (http://rayyan.qcri.org)^12^ was used to organize and review articles. Two search strategies of Google Scholar, Pubmed, Embase, and Web of Science were used to maximize identified articles. The first search was “osseointegration” or “osseointegrated” or “bone anchored prosthesis” AND “known osseointegration author last name.” Specific authors searched included Branemark, Hagberg, Aschoff, Grundei, Al Muderis, Tetsworth, Hoellwarth, Bloebaum, Bachus, Leijendekkers, Frolke, van de Meent, Pendegrass, Rozbruch, and Reif, Kang. The second search was (osseointegration OR osseointegrated OR “bone anchored prosthesis”) AND (rehabilitation OR rehabilitated OR “physical therapy”) AND (lower extremity OR femur OR femoral OR tibia OR tibial OR transfemoral OR transtibial). Selected articles’ references were reviewed to identify their foundational literature and recursively tracked further back using successive articles’ references. Additionally, Google Scholar’s “cited by” feature was also used to discover newer articles potentially relevant by virtue of citing the article of interest.

### Selection of Studies

The software removed duplicate articles. A single author screened all remaining articles identified in the primary searches. Articles whose abstract reported primary lower extremity osseointegration data were selected for full text review. Articles were ultimately selected for inclusion if there was any description of postoperative physical therapy or rehabilitation (such as how patients were rested, loaded, and advanced to ambulation). In order to avoid potential confusion of rehabilitation strategies without proof of suitability, articles not reporting patient care outcomes were excluded (such as study proposals without patient data or biomechanical simulations).

### Data Extraction

The data were organized using Google Sheets (Alphabet Inc, Mountain View, CA). Two authors reviewed each included article’s full text to extract the postoperative rehabilitation description, then consolidated key principles: time between surgical stages (when applicable), time from externalization surgery until physical therapy, time until loading initiation, maximum first load, load progression pattern, and time from externalization to independent ambulation.

### Data Analysis and Presentation

The primary aim of this investigation was to identify commonalities and differences among published protocols. It is emphasized that this scoping article does not aim to rank or compare aspects such as the appropriateness, success, or popularity of the rehabilitation protocols; only to present the spectrum of principles to bring awareness and to better facilitate future research specifically of the rehabilitation of this patient population. Therefore, the Results section presents each major category of the rehabilitation process organized by Group or Medical Center in order to aid contextualization of protocol differences, and summarizes the spectrum of data with little or no assessment or statistical comparison of eventual mobility or QOL outcomes. Not only was analysis or comparison of the protocols not the aim of this study (recent review of patient outcomes are already published^9^), but also the published literature does not allow such comparison to be performed with specific regard to rehabilitation protocol. The secondary aim of this study was to suggest themes and focus areas which may best streamline future TOFA rehabilitation research, with the intent to inspire specific research of the techniques, principles, and practicalities of pre- and postoperative rehabilitation of this patient population.

## RESULTS

**Figure 1:**
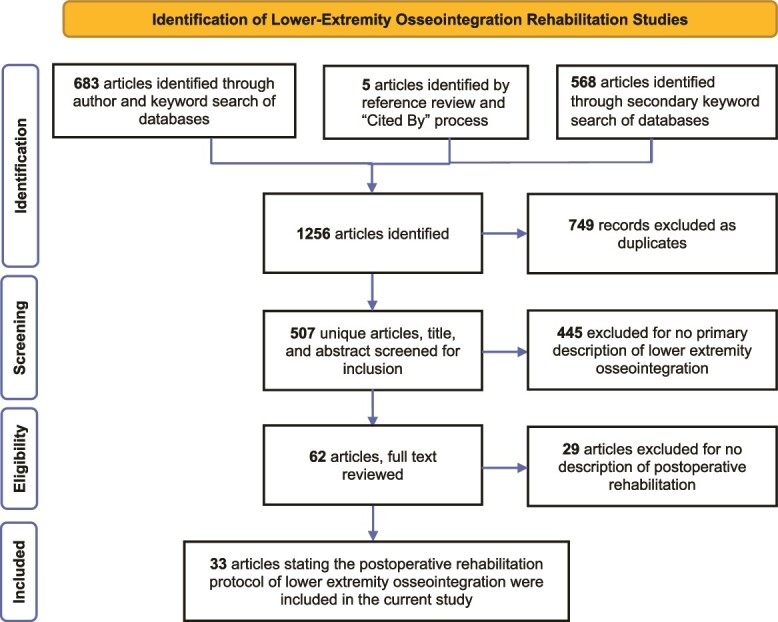
Article Screening Process.

Figure 1 shows the article screening process which is detailed in the legend. This yielded 35 articles for inclusion. The following implant systems and patients were described: Osseointegrated Prostheses for the Rehabilitation of Amputees (OPRA, Integrum, Sweden), transfemoral only (except one tibia case report); endo–exo implant (ESKA Orthopaedic Handels, Germany) which became integral leg prosthesis (ILP, Orthodynamics, Germany); and osseointegrated prosthetic limb (OPL, Permedica Manufacturing, Italy) for transfemoral and transtibial amputations. A custom press-fit prosthetics (Signature Orthopaedics, Australia) were used in patients with transfemoral amputations, osseointegration femoral implant (OFI, OTN implants, Netherlands), and osseointegration tibia implant (OTI, OTN implants, Netherlands). It is very important to emphasize, however, that articles and protocols that were used for differing level**s** (femur or tibia) or amputation etiology did not specify any difference in the rehabilitation regimen based on these factors.

### Primary Aim of Study: Assessment of Protocols

**Figure 2:**
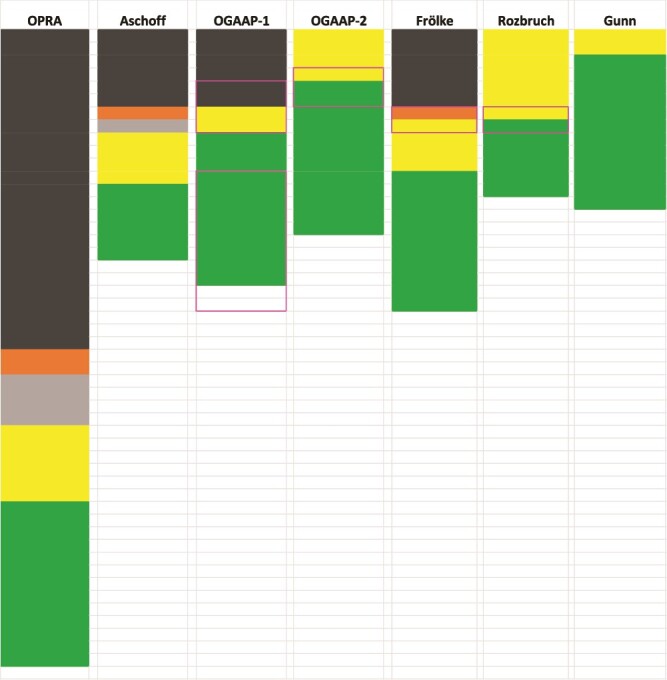
Graphical Illustration of Post-operative Lower Extremity Rehabilitation Following Osseointegration Surgery Protocol Timelines. The horizontal axis represents the different rehabilitation protocols (when named) or the representative surgeon. The vertical axis enumerates the number of weeks that each rehabilitation stage consists of. All authors followed the same order of recovery as stated in the text. Colors indicate the different phases. When not mentioned in the protocol (or if less than 1 week in length), the phase (color) is omitted from this overview schematic. Black—waiting period between surgical stages; orange—resting period; gray—physical therapy with no weight bearing; yellow—training prosthetic period; green—aided walking with a full-length prosthetic. The pink outlines represent the range during which transition to the next stage occurs, when specified by the author. OGAAP-1 = Osseointegration Group of Australia’s Accelerated Protocol 1; OGAAP-2 = Osseointegration Group of Australia’s Accelerated Protocol 2; OPRA = Osseointegrated Prostheses for the Rehabilitation of Amputees.

The primary aim of this scoping review was to identify commonalities and differences among protocols. Nine general groups of authors and protocols were identified: Brånemark (OPRA), Aschoff, Al Muderis (Osseointegration Group of Australia’s Accelerated Protocol 1 [OGAAP-1] and Osseointegration Group of Australia’s Accelerated Protocol 1 [OGAAP-2]), Frölke, Rozbruch, Davis-Wilson, McMenemy, Di Paolo, and Gunn. The most important commonality identified by the article review process was that all protocols had the same goal progression, as detailed in Table 1, illustrated in Figure 2, and summarized as subsequently described. Since no studies evaluated or compared outcomes based on rehabilitation protocol, the most significant comparable metric appeared to be time from surgery to independent ambulation; therefore, the most impactful difference among protocols is whether there is one or two surgical stages and the time between the two stages. Surgery is always completed first, either with two stages (intramedullary stem placement followed by separate insertion of the transcutaneous adapter later) or one stage (both portions in a single surgery).^13–23^^,^^29–32^^,^^39–44^^,^^47^^,^^51^^,^^52^ Some authors include a resting period following externalization. All authors progressively load patients toward full weight bearing, but with different patterns as detailed in Table 2. All rehabilitation descriptions conclude with independent full weight bearing ambulation. The following sections summarize each common element of goal progression, clarifying the variation among protocols.

**Table 1:** Rehabilitation Protocols Used in All Analyzed Studies, Grouped by Anatomic Level*^a^*

First Author	Year	Transtibial/Transfemoral	Amputation Etiology	Implant “Used” [IPL, OPL...]	Group	Time From Stage 1 to Stage 2	Time From Final Surgery to Initiating Physical Therapy	Time to Load Following Stage 2	Time W/Training Prosthetic/Loading Exercises Prior to Definitive Prosthetic	Double Crutch Support	Single Crutch Support	Referenced Previously Published Rehabilitation Protocol
						**Transfemoral**						
Hagberg et al^13^	2009	Transfemoral—100	Trauma (67) | tumor (21) | other (12)	OPRA	Brånemark R	6 mo	2 wk	4–6 wk	5–6 wk	3 mo	Unstated	Non-referenced
Ferencz et al^14^	2009	Transfemoral—2	Other	OPRA	Brånemark R	6 mo	Unstated	Unstated	Unstated	Unstated	Unstated	Non-referenced
Aschoff et al^15^	2011	Transfemoral—39	Trauma | tumor | infection	Endo–exo	Aschoff	6 wk	7–10 d	8 wk	Unstated	Unstated	Unstated	Non-referenced
Van de Meent^16^	2013	Transfemoral—22	Trauma (20) | tumor (2)	ILP	Frölke	6 wk	Unstated	2 wk	2 wk	Unstated	Unstated	Non-referenced
Brånemark et al^17^	2014	Transfemoral—51	Trauma (33) | tumor (12) | other (6)	OPRA	Brånemark R	6 mo	Unstated	Unstated	Unstated	Unstated	Unstated	Hagberg et al^13^
Juhnke et al^18^	2015	Transfemoral—69	Trauma (51) | tumor (7) | infection (1) | burn (1) | other (7)	Endo–exo	Aschoff	6 wk	Immediately	Unstated	Unstated	Unstated	Unstated	Non-referenced
Schalk et al^19^	2015	Transfemoral—1	Trauma (1)	Custom	Aschoff	6 wk	1 wk	1 wk	Unstated	Unstated	Unstated	Non-referenced
Al Muderis et al^20^	2016	Transfemoral—50	Trauma (35) | tumor (5) | congenital (8) | other (2)	ILP + OPL	Al Muderis	4–8 wk	3 d	3 d	11 d	6 wk	6 wk	Non-referenced
Leijendekkers^21^	2017	Transfemoral—1	Trauma (1)	EEFP	Frölke	6 wk	2 wk	2 wk	2 wk	3.5 wk	Unstated	Hagberg et al^13^ Aschoff et al^15^
Al Muderis et al^22^	2018	Transfemoral—37	Trauma (37)	ILP + OPL	Al Muderis	6 wk	3 d	3 d	11 d	6 wk	6 wk	Al Muderis et al^20^
Matthews et al^23^	2019	Transfemoral—18	Trauma (18)	OPRA	Brånemark R	6 mo	0 wk	6 wk	2–3 mo	Unstated	Unstated	Hagberg et al^13^
Hansen et al^24^	2019	Transfemoral—17	Trauma (7) | tumor (4) | infection (2) | vascular (1) | other (4)	OPRA	Hansen	6 mo	Unstated	6 wk	Unstated	Unstated	Unstated	Hagberg et al^13^
Reetz et al^25^	2020	Transfemoral—39	Trauma (29) | tumor (6) | infection (3) | other (1)	ILP	Frölke	6–8 wk	2 wk	2 wk	2 wk	Unstated	Unstated	Al Muderis et al^26^ Leijendekkers et al^21^
McMenemy et al^27^	2020	Transfemoral—7	Trauma (7)	OPL	McMenemy	One stage/2 stages (n/a)	Unstated	3 d	12 wk	Unstated	Unstated	Al Muderis et al^28^
Hoellwarth et al^29^	2021	Hip—1	Trauma (1)	ILP	Al-Muderis	One stage	3 d	Unstated	Unstated	Unstated	Unstated	Al Muderis et al^20^^,^^28^
Akhtar et al^30^	2022	Transfemoral—10	Other (10)	ILP + OPL	Al Muderis	One stage	3 d	3 d	2–3 wk	Unstated	Unstated	Al Muderis et al^20^^,^^28^
Krause et al^31^	2022	Transfemoral—1	Other (1)	Endo–exo	Aschoff	6 wk	2 d	2 d	Unstated	6 wk	Unstated	Non-referenced
Gun et al^32^	2022	Transfemoral—1	Trauma (1)	Unstated	Gunn	One stage	3 d	3 d	12 d	6 wk	6 wk	Al Muderis et al^28^
Gaffney et al^33^	2022	Transfemoral—4	Trauma (2) | tumor (1) | vascular (1)	OTN	Davis-Wilson	6 wk	2 d	Unstated	Unstated	Unstated	Unstated	Leijendekkers et al^21^ Brånemark et al^34^ Vertriest et al^35^
Davis-Wilson et al^36^	2023	Transfemoral—4	Unstated	OTN	Davis-Wilson	6–8 wk	2 d	Unstated	Unstated	Unstated	Unstated	Non-referenced
Gaffney et al^37^	2023	Transfemoral—10	Trauma (4) | tumor (5) | vascular (1)	OTN	Davis-Wilson	6 wk	2 d	Unstated	Unstated	Unstated	Unstated	Brånemark et al^34^ Leijendekkers et al^21^
Di Paolo et al^38^	2023	Transfemoral—1	Trauma (1)	OTN	Di Paolo	6 wk	15 d	Unstated	Unstated	Unstated	Unstated	Non-referenced
						Transtibial						
Khemka et al^39^	2015	Transtibial—4	Trauma (1) | infection (4)	Custom	Al Muderis	4–6 wk	1 d	Unstated	Unstated	6 k	6 wk	Al Muderis et al^20^^,^^28^
Atallah et al^40^	2017	Transtibial—5	Vascular (5)	OPL + ILP	Al Muderis	One stage/2 stages (6–8 wk)	Unstated	Unstated	2–4 wk	Unstated	Unstated	Al Muderis et al^20^ Leijendekkers et al^41^
Akhtar et al^42^	2021	Transtibial—6	Trauma (1) | vascular (5)	OPL + ILP	Al Muderis	Unstated	3 d	3 d	Unstated	Unstated	Unstated	Al Muderis et al^20^ Leijendekkers et al^41^
Gstoettner et al^43^	2021	Transtibial—1	Trauma (1)	OPRA	Brånemark R	3 mo	3 wk	3 wk	Unstated	Unstated	Unstated	Non-referenced
						Both						
Juhnke et al^44^	2015	Transfemoral—67 | Transtibial—7	Trauma (56) | tumor (7) | other (11)	Endo–exo implant	Aschoff	6 wk	3 d	Unstated	Unstated	Unstated	Unstated	Non-referenced
Leijendekkers et al^45^	2019	Transfemoral—31 | Transtibial—9	Trauma (55) | tumor (18) | vascular (13) | other (15)	OPL + ILP + custom	Frölke	6–8 wk	1 wk	1 wk	Unstated	Unstated	Unstated	Leijendekkers et al^21^^,^^41^
Atallah et al^46^	2020	Transfemoral—66 | Transtibial—20 | Through knee—3	Trauma (50) | vascular (12) | infection (12) | tumor (8) | congenital (3) | other (8)	OFI-C + OFI - Y + OTI	Frölke	One stage/2 stages (6–8 wk)	1 wk—2 stage | 3 wk—single stage	1 wk—2 stage | 3 wk—single stage	Unstated	Unstated	Unstated	Leijendekkers et al^21^^,^^45^
Reif^47^	2021	Transfemoral—18 | Transtibial—13	Trauma (22) | chronic periprosthetic infection (2) | Necrotizing fasciities (1) | vascular (2) | neurological injury (3) | deformity (1)	OPL + custom	Rozbruch	One stage/2 stages (8 wk)	1 d	1 d	6–8 wk	Unstated	Unstated	Non-referenced
Haidary et al^48^	2023	Transfemoral—3 | Transtibial—5	Burn (5)	ILP + OPL	Al Muderis	One stage	Unstated	Several days	4–6 wk	Unstated	Unstated	Non-referenced
Davis-Wilson et al^49^	2023	Transfemoral—9 | Transtibial—3	Trauma | congenital | cancer-related	Press-fit - type unstated	Davis-Wilson	One stage—transtibial/2 stages (6–8 wk) —transfemoral	2 d—transfemoral | 6 wk—transtibial	2 d—transfemoral | 6 wk—transtibial	Unstated	Unstated	Unstated	Non-referenced
Atallah et al^50^	2023	Transfemoral—72 | Transtibial—8	Trauma (47) | vascular (8) | infection (9) | tumor (12) | congenital (1) other (3)	Endo–exo + ILP + OPL	Frölke	6–8 wk	1 wk	Unstated	Unstated	Unstated	Unstated	Leijendekkers et al^21^^,^^45^

^a^ EEFP = endo–exo femur prosthesis; ILP = integral limb prosthesis; n/a = not available; OFI-C = osseointegration femur implant—C; OFI-Y = osseointegration femur implant—Y; OPL = osseointegrated prosthetic limb; OPRA = Osseointegrated Prostheses for the Rehabilitation of Amputees; OTI = osseointegration tibia implant; OTN = Badal X Implant.

### Surgical Stages

The rehabilitative experience for patients undergoing lower extremity osseointegration begins with surgery to place the osseointegrated prosthesis anchor. The first published protocol was written by Brånemark’s group (OPRA), and specified two surgical stages separated by 6 months with a detailed therapy regimen between the surgical stages.^13^ Most other groups have used a two-surgery protocol but have varied the time between stages: Aschoff (6 weeks), Al Muderis (OGAAP-1, 4–6 weeks), David-Wilson (6–8 weeks), Di Paolo (6 weeks), Frölke (6–8 weeks), and three patients from Rozbruch (8 weeks). Some groups have written protocols featuring a single surgical stage: Al Muderis (OGAAP-2), Rozbruch, and Gunn. As shown in Figure 2, the time between Stage 1 and Stage 2 has the largest impact regarding when patients are expected to achieve independent ambulation following surgery, particularly for the protocols which exceed 8 weeks between stages.

### Externalization Surgery to Therapy Commencement

Following externalization of the implant, whether at the second surgery or the single one, most groups specify some duration until therapy is initiated, or re-initiated. The Brånemark (OPRA)-based protocols ranged from within the first week^23^ to two weeks.^13^ The Aschoff-based protocols ranged from immediate^44^ to 8 weeks.^15^ Al Muderis (OGAAP-1^22^ and OGAAP-2^30^) consistently recommended 3 days following final surgery. Frölke and Leijendekkers reported 1 week^51^ or 2 weeks^21^, respectively. Reif reported waiting 1 day before loading,^47^ and Gunn reported 3 days.^32^

### Progressive Loading

In addition to the time from final surgery to loading, there was variation regarding how much to start and progress loading. The Brånemark (OPRA) protocols generally recommended starting at 20 kg and progressing 10 kg/week^13^^,^^23^ but another case report of a tibia salvage situation started the loading at 10 kg.^43^ Aschoff reported loading starts at 10 kg and although exact progression was not stated, full body weight was expected within 2–4 weeks.^15^ Whether one or two stages, Al Muderis reported starting at 20 kg and progressing 5 kg per week to 50 kg or half body weight.^20^^,^^28^ Frölke reported starting at 50% body weight and achieving full weight by week 4.^16^ Rozbruch recommended starting at 9 kg and progressing 2.3 kg every other day to full body weight. Gunn followed the OGAAP-1 loading protocol.

**Table 2:** Weight Progression of Rehabilitation Protocols in All Analyzed Studies, Grouped by Anatomic Level*^a^*

First Author	Year	Implant “Used” [IPL, OPL...]	Group	Max First Load (kg) (X = Relative to Body Weight)	Times Per Day	Length of Time (min)	Load Progression	Time to Full Weight Following Externalization
				**Transfemoral**				
Hagberg et al^13^	2009	OPRA	Brånemark R	20	2	30	10 kg/wk	6 mo
Ferencz et al^14^	2009	OPRA	Brånemark R	Unstated	Unstated	Unstated	Unstated	Unstated
Aschoff et al^15^	2011	Endo–exo	Aschoff	10 kg	Unstated	Unstated	Unstated	2–4 wk
Van de Meent^16^	2013	ILP	Frölke	50% body weight	Unstated	Unstated	Full weight wk 4	4 wk
Brånemark et al^17^	2014	OPRA	Brånemark R	Unstated	Unstated	Unstated	Unstated	Unstated
Juhnke et al^18^	2015	Endo–exo	Aschoff	Unstated	Unstated	Unstated	Unstated	4–6 wk
Schalk et al^19^	2015	Custom	Aschoff	Unstated	Unstated	Unstated	Unstated	4 wk
Al-Muderis et al^20^	2016	ILP + OPL	Al Muderis	20 kg	2	20	5 kg/d to 50 kg or 1/2 body weight	Unstated
Leijendekkers^21^	2017	EEFP	Frölke	Unstated	2 - > 3	30 - > 60 w/full weight bearing	Unstated	As low as 4 wk
Al-Muderis et al^22^	2018	ILP + OPL	Al Muderis	20 kg	2	20	5 kg/d to 50% body weight	Unstated
Matthews et al^23^	2019	OPRA	Brånemark R	20 kg	2	15–30	10 kg/wk to full weight	60–90 d
Hansen et al^24^	2019	OPRA	Hansen	20 kg	Unstated	Unstated	10 kg/wk	Up to 6 mo
Reetz et al^25^	2020	ILP	Frölke	Unstated	Unstated	Unstated	_____ to full body weight	Unstated
McMenemy et al^27^	2020	OPL	McMenemy	20 kg	Unstated	Unstated	5 kg/d to 50 kg or 50% body weight	12 wk
Hoellwarth et al^29^	2021	ILP	Al-Muderis	Unstated	Unstated	Unstated	Unstated	Unstated
Akhtar et al^30^	2022	ILP + OPL	Al Muderis	Unstated	Unstated	Unstated	_____ to half body weight	4–6 wk
Krause et al^31^	2022	Endo–exo	Aschoff	5–10 kg	Unstated	Unstated	Unstated	6 wk
Gun et al^32^	2022	Unstated	Gunn	20 kg	2 - > 3	30	5 kg/d to 80%–90% body weight	12 wk
Gaffney et al^33^	2022	OTN	Davis-Wilson	Unstated	Unstated	Unstated	Unstated	Unstated
Davis-Wilson et al^36^	2023	OTN	Davis-Wilson	Unstated	Unstated	Unstated	Unstated	Unstated
Gaffney et al^37^	2023	OTN	Davis-Wilson	Unstated	Unstated	Unstated	Unstated	Unstated
Di Paolo et al^38^	2023	OTN	Di Paolo	Unstated	Unstated	Unstated	Unstated	Unstated
				**Transtibial**				
Khemka et al^39^	2015	Custom	Al Muderis	20 kg	2	20	5 kg/wk to 50 kg or 1/2 body weight	Unstated
Atallah et al^40^	2017	OPL + ILP	Al Muderis	5 kg	Unstated	Unstated	5 kg/d to 50 kg or 1/2 body weight	4–6 wk
Akhtar et al^42^	2021	OPL + ILP	Al Muderis	Unstated	Unstated	Unstated	_____ to full weight	Unstated
Gstoettner^43^	2021	OPRA	Brånemark R	10 kg	Unstated	Unstated	_____ to full body weight	Unstated
				**Both**				
Juhnke et al^44^	2015	Endo–exo implant	Aschoff	Unstated	Unstated	Unstated	Unstated	3 d
Leijendekkers et al^45^	2019	OPL + ILP + custom	Frölke	Unstated	Unstated	Unstated	Unstated	4 wk - transtibial | 11 wk - transfemoral
Atallah et al^46^	2020	OFI-C + OFI - Y + OTI	Frölke	Unstated	Unstated	Unstated	_____ to full body weight	4 wk - transtibial | 11 wk - transfemoral
Reif et al^47^	2021	OPL + custom	Rozbruch	9 kg	Unstated	Unstated	2.3 kg/2 d to full weight	6–8 wk
Haidary et al^48^	2023	ILP + OPL	Al Muderis	Unstated	Unstated	Unstated	_____ to half body weight	12 wk
Davis-Wilson et al^49^	2023	Press-fit	Davis-Wilson	Unstated	Unstated	Unstated	Unstated	Unstated
Atallah et al^50^	2023	Endo–exo + ILP + OPL	Frölke	Unstated	Unstated	Unstated	_____ to full body weight	11 wk

^a^ EEFP = endo–exo femur prosthesis; ILP = integral limb prosthesis; OFI-C = osseointegration femur implant **- C**; OFI-Y = osseointegration femur implant - Y; OPL = osseointegrated prosthetic limb; OPRA = Osseointegrated Prostheses for the Rehabilitation of Amputees; OTI = osseointegration tibia implant; OTN = Badal X Implant.

### Progression Through Gait Training

The description of gait training had particular variability among protocols (Table 2). Brånemark (OPRA) recommended beginning gait training at 12 weeks and using two crutches for an additional 3 months.^13^ Aschoff protocols did not specify gait progression except for reconstruction of periprosthetic fractures, which restarted walking at 6 weeks with two crutches for a subsequent 6 weeks.^31^ OGAAP protocols were unclear exactly when to start gait training, but to use two crutches for 6 weeks followed by one crutch for an additional 6 weeks.^20^ Leijendekkers detailed the timing for gait progression in a case report, stating initiation of walking 4 weeks after externalization and the use of two crutches for 3.5 weeks.^21^ Gunn followed the Al Muderis gait progression. Protocols did not express performance measures for progression or regression (such as steps achieved without tripping or distance walked per time) but rather only a timeline.

### Secondary Aim of Study: Opportunities of Improving Rehabilitation Through Research

The secondary aim of this scoping review was to propose strategies to improve TOFA rehabilitation research. While all articles identified timing for phase progression, none supported these recommendations with basic science principles or provided clinical data validating the recommendations. Additionally, no articles stated the percentage of patients who achieved each phase or completed care as scheduled; stated another way, no article truly studied the success or suitability of the protocol. None provided specific qualitative or quantitative measures such as patient sex, age, bone density, implant size, or amputation level to guide the determination of rest time before weight bearing or the rate of weight progression. Some authors changed the specific timing of phases versus foundational protocols, but provided no reasoning for such changes. Further, no articles reported challenges or workarounds to ultimately achieve success. The rehabilitation protocols formally concluded with achievement of independent ambulation, nothing more. A few articles by Al Muderis, Leijendekkers, and the Food and Drug Administration (FDA)-associated OPRA protocol stated that, following achievement of independent ambulation, additional gait training focusing on fall prevention and management, balancing, and walking on sloped terrain could be prescribed to patients, but did not state the indications for doing so nor describe the specific training or maneuvers.^21^^,^^22^^,^^53^ It is important to re-emphasize: no investigations differentiated rehabilitation planning based on patient characteristics (such as age, sex, femur vs tibia amputation level, amputation etiology, or whether they were an existing amputee or simultaneous amputation with osseointegration) or implant characteristics (such as diameter or length). Proposals to address each of these critiques will be presented in the Discussion.

## DISCUSSION

There were two goals of this study. Primarily, to determine the commonalities and differences among published TOFA rehabilitation routines. Secondarily, to suggest possible strategies to optimize patient outcomes through rehabilitation protocol optimization. Regarding the primary goal, the most notable commonality was limited details of rehabilitation following TOFA; usually the rehabilitation protocol was one paragraph. Articles were consistent in the order of goal progression from surgery to gradual weight bearing, with full independent weight bearing being the final rehabilitation goal. The most notable difference among protocols was whether one or two surgical stages were used (major impact on formal rehabilitation time) and the speed of goal progression (lesser impact). Regarding the secondary aim, the most notable limitation of current research of rehabilitation post-osseointegration surgery is that no investigations differentiated rehabilitation planning based on patient characteristics (such as age, sex, femur vs tibia amputation level, amputation etiology, or whether they were an existing amputee or simultaneous amputation with osseointegration) or implant characteristics (such as diameter or length). None reported the actual ability to achieve the proposed goals and timing, or the challenges and their workarounds during rehabilitation.

The most important impression we hope to share to readers is the recognition of the acute need for research directly studying how to optimize the postoperative rehabilitation of patients with lower extremity osseointegration. Nearly all osseointegration-related literature focuses on surgery, technology, basic science, or surgical complications of TOFA. The rehabilitation strategies and challenges are under-represented. Therefore, we believe the paramount recommendation to optimize patient recovery is to specifically study TOFA rehabilitation techniques and their impact on patient outcomes.

Understanding challenges to the study of TOFA rehabilitation is important in overcoming those challenges. The following themes seem relevant. First, TOFA is a relatively new procedure and, until recent FDA approval for one implant, was available in only a few practices worldwide. Patients often live very far from a TOFA center and may return home relatively soon after surgery, so few physiatrists and physical therapists may have concentrated long term and high volume experience treating patients following TOFA. Similarly, social awareness of TOFA remains relatively low; patients may feel socially marginalized following amputation,^54^ but given current social media trends promoting body image empowerment this may improve.^55^ In contrast, social awareness and also financial investment is much higher for some situations which have established benefit from specific postoperative physical therapist protocols, namely total hip and knee arthroplasty.^56^ Reasons for the social and financial differences may include the higher incidence of arthroplasty procedures in the overall population,^57^ the associated larger financial incentive to return the patients to activities or employment,^58^ and the public awareness of the lifestyle benefits of arthroplasty.^59^ These statements are written not to attack the excellent advances resulting from research into arthroplasty-specific physical therapy, but rather to highlight the potential role for similar treatment-specific research into the rehabilitation of patients following TOFA.

The financial cost of TOFA may be another reason the surgery and associated physical therapy is not more broadly available. Given the immense demand for and resultant economy related to joint replacement, hospitals and implant companies understand the financial benefit of improved outcomes.^60^ Limb loss may traditionally be regarded as certain financial loss,^61^ so there may be a bias toward “minimizing losses” rather than “maximizing return on investment”.^62^ However, financial estimates suggest TOFA to be cost-advantageous versus socket prostheses over time despite a higher initial cost (due to initial surgery and implant costs),^63^ notably because it is unnecessary to remake sockets.^64^ This financial benefit is in addition to the mobility and QOL benefits.^9^ More studies of employment potential or other long term care needs for patients following TOFA will further elucidate important financial aspects. Physical therapy is essential to safe and effective patient recovery following osseointegration, and therefore it is important to maximize the physical therapy benefit by researching optimal protocols and techniques. As a side benefit, attention given to rehabilitation protocols for the lower extremities will likely optimize the development of protocols for upper extremity osseointegration, the demand for which may occur soon given the rapid improvement of neural interface technology and prostheses.^65^

Recognition of the specific need for physical therapy following osseointegration may also be lacking. Over the last several decades, postoperative performance and satisfaction following total hip and knee arthroplasty have improved not only because of technical and technique improvements,^66^ but also because of persistent scientific analysis of the outcomes of rehabilitation protocols.^67^ For example, studies have identified optimal rehabilitative techniques for patients who have additional mobility challenges such as stroke,^68^ and also the benefit of function-directed physical therapy following joint replacement.^69^ Investigations into restoring lifestyle activities as specific as playing golf have become so common as to have systematic reviews.^70^ This statement is not intended to suggest that only golf-centric rehabilitation ought to be pursued for osseointegrated patients, or to deride the research of golfing for arthroplasty patients. Instead, the intent is to emphasize that therapy directed at returning patients to recreational and lifestyle activities beyond simple ambulation may be one approach to optimizing post-amputation mobility, and with it improve the healthcare system’s return of financial investment in that patient’s care. Further, considering that greater physical activity reduces diabetes-related lower extremity ulcer rate,^71^ the improved mobility conferred by osseointegration—a safe option for diabetic and vascular populations^42^^,^^72^—may prevent additional amputations,^73^ improving patient health and reducing costs. Further, for patients who sustain amputations when otherwise young and healthy, preventing subsequent disability and setting motivating expectations to return to a career can maximize the person’s and also society’s financial future.^74^ For these reasons, attention to the rehabilitation of osseointegrated amputees is necessary.

With the aforementioned reasons for increased TOFA rehabilitation investigation identified, the following suggestions are offered in an effort to encourage and guide potential research. The first recommendation is to investigate the feasibility of and rationale behind achieving the goals stated in protocols. For example, the adverse events and “success” rate for various phases of protocols should be investigated. The following five specific topics seem both relevant and accessible to investigate. The true range of time following surgery that patients have actually started loading (not just the stated recommendation, but the actual number of days postoperatively patients in fact loaded) and the association with their mobility outcomes at three or six or twelve months. The percentage of patients achieving the recommended loading weight (such as 10 kg) on the intended day, or who cannot and why not (perhaps due to issues such as pain or infection). The rate of adverse events (such as falls) and how they are avoided. The strategies utilized and success rates when patients have loading pain. Any elucidation of demographic, surgical, institutional, or other identifiable factors associated with patient success or challenge may help optimize postoperative rehabilitation: specifically, the level of amputation (transfemoral vs transtibial) or experience with prior socket prosthesis use likely have a substantial impact on the progression of mobility goals.^41^^,^^51^

The second recommendation is to more thoroughly explore amputee-centric rehabilitation of performance deficits. While simple ambulation was an appropriate goal to set when osseointegration was just getting started, it is no longer ambitious enough for many patients. As with any patient population, amputated patients may have deficits that are common among the population (such as hip flexion contractures for transfemoral amputees,^75^ knee flexion contractures for transtibial amputees,^76^ or certain gait changes^77^) and/or also individual deficits that may be related to the etiology of their amputation (such as arthritis following post-traumatic amputation) or simply unique to themselves. Patients must substantially recalibrate their stance, gait, and other activities^21^ for safe mobility. Furthermore, it is imperative to understand what physical therapy activities present increased risk of injury (for example, standing on a BOSU ball may lead to fall and periprosthetic fracture), so they can be avoided or more safely pursued.^78^ Other examples may include exploring the efficacy and safety of vibrating platforms in improving balance, muscle strength, and bone density,^79^ or the safety of resistance bands on the actual prosthesis (not just on the bone).^80^ Additionally, evaluating specific physical therapy regarding how to fall, don a prosthesis from the ground, rise following a fall, and further training for fall prevention, balance, and walking on sloped ground, could help patients feel safer ambulating in more challenging environments.^21^^,^^22^

A third recommendation for study is the use of new technological advancements to better understand patient mobility change following osseointegration. Formal laboratory gait analysis can provide objective quantitative data regarding weaknesses or imbalances in gait, muscle activity, and ground-reaction forces during ambulation.^16^ Understanding these aspects may optimize rehabilitation strategies to overcome challenges and reduce risk of secondary musculoskeletal conditions (like back pain, instability, and compensatory movements during ambulation).^41^ Non-formal, markerless gait analysis may be a more affordable and accessible alternative.^81^ This can allow better analysis of patient motion in more places such as on the beach or climbing boulders or skiing. Interpretation of that motion can guide rehabilitation strategies by identifying favorable or pathologic accommodations. Another exciting realm to monitor rehabilitation is the use of activity trackers.^82^ Such devices can provide more reliable data regarding wear time, step count, and other aspects of mobility than patient surveys. Built-in monitoring for total knee arthroplasty has improved early prediction of outcomes^83^^,^^84^; a monitor for osseointegrated prostheses may help in similar ways, and may also help detect patient falls. Connecting an activity tracker to a smart phone may additionally improve prosthesis engagement and use via gamification,^85^ which has proven very beneficial for multiple musculoskeletal rehabilitation situations.^86^^,^^87^ Helping patients experience therapy in a way that feels more entertaining or engaging may quicken their acquisition of skills or deepen their mastery of those skills.^88^ Particularly for patients who may feel isolated, gamification can improve their mental wellbeing.^89^

There are limitations to consider for this study. Scoping reviews by definition aim to provide breadth over depth; however, we believe we have also reviewed a substantial amount of the currently available depth regarding this topic. We believe that a scoping review is the appropriate review of this topic, given the current quantity and quality of literature. Indeed, this study highlights the need for specific rehabilitation research. It is emphasized: this study does not aim to compare or “rank” the “goodness” of the various rehabilitation protocols, in part specifically because the available outcomes do not have evidence closely associating the mobility, QOL, or adverse outcomes with the rehabilitation protocols. An important strength of this study is the depth and breadth of article inclusion. The only other review article of rehabilitation following TOFA surgery^90^ evaluated 10 articles, whereas 35 were included in the current review. Further, we present figures and tables which improve the visual interpretation of the different rehabilitation timings and principles that were also provided in textual description.

## CONCLUSIONS

All protocols for rehabilitation post-osseointegration surgery have the same order of goal progression: from surgery to gradual weight bearing with a final goal of independent ambulation. There are no studies evaluating patient rehabilitation challenges or variation in achieving rehabilitation progression. There is an acute need for research directly investigating how to optimize the postoperative rehabilitation of patients with lower-extremity osseointegration. It is hoped that suggestions offered within this review study can guide such research.

## Data Availability

This scoping review is derived from publicly available online sources. All articles analyzed in this scoping review are accessible through the academic databases reported in the methods section.
